# Supplementary material for: Global metabolic interaction network of the human gut microbiota for context-specific community-scale analysis
Source: Nat Commun. 2017 Jun 6;8:15393. doi: 10.1038/ncomms15393 (PMC5467172; doi:10.1038/ncomms15393)
Supplement: Supplementary Information — Supplementary Methods [file ncomms15393-s1.pdf]

Supplementary Methods

Identification of metabolic cliques

We introduce the concept of a metabolic clique, i.e., a group of microbial species that import, export, or degrade a metabolite or macromolecule. Each compound (metabolite or macromolecule) and microbial activity (import, export, or degradation) defines a single metabolic clique (e.g., glucose importers, butyrate exporters, or cellulose degraders). To consolidate metabolic cliques that have almost identical species compositions, we conducted a complete-linkage hierarchical clustering of metabolic cliques by unifying pairs of metabolic cliques with large Jaccard indices (≥ 0.8) between their species memberships. The resulting metabolic cliques were used for further analyses.

Identification of microbial entities differentially abundant or scarce in T2D

For the male, mid-age, normal weight cohort, we found microbial species with significant differences in their abundances between T2D and control samples, by applying the Wilcoxon rank-sum test, and then the Benjamini–Hochberg false discovery rate (FDR) correction for multiple testing (FDR < 0.1). Here, we tested only for species with practically meaningful abundances (median abundance from either T2D or control samples of ≥ 0.001%. The same threshold was used when calculating a microbial metabolic influence W_{ij}, as explained below). As a result, we identified species differentially abundant or scarce in T2D (in other words, differentially scarce or abundant in control, respectively). We applied the same methods when identifying groups of microbes (genera or metabolic cliques, each comprising multiple species), which are differentially abundant or scarce in T2D. In this case, the abundance of each group was defined as the total abundance of all species in the group.

Identification of the microbial metabolic influence network and its influencers

1. Overview

For the male, mid-age, normal weight cohort, we converted the bipartite network (NJS16) composed of microbial species and metabolic compounds into the unipartite network of microbial entities, i.e., species, genera, and metabolic cliques differentially abundant or scarce in T2D compared to control. This network is called herein a microbial metabolic influence network, which can be constructed by the following two broad steps:

(*i*) Given microbial entities *i* and *j*, we first estimate the potential metabolic influence of one entity *i* upon another entity *j*'s growth (and vice versa). The sign of this quantity can indicate whether the metabolic influence is positive (growth-promotive) or negative (growth-inhibitive).

(*ii*) With weight proportional to the quantity above, we assign a directed link from entity *i* to entity *j*. For every pair of differentially abundant or scarce microbial entities, we assign those links. Among those links, we consider only the links that account for actual microbial abundance changes between control and T2D subjects.

Of note, one entity (differentially abundant or scarce in T2D) can be a subset or element of another entity, or can have a large intersection with it, in terms of their species members. This indicates that a certain entity may exhibit differential abundance, not because of its own nature, but because of its species overlapping with those of another differentially abundant or scarce entity. To prevent such redundant effects, the influence network primarily includes the entities that still exhibit differential abundance under the "subtraction" of the other entities' species; henceforth, this examination with the subtraction is called herein the redundancy test for differential abundance.

When implementing step (*i*), the following multiple issues should be carefully addressed: microbe-microbe metabolic interactions, microbe-host metabolic interactions, macromolecule degradation by

microbes, and metabolic compounds derived from dietary or abundant endogenous sources. In step (*i*), we quantify the potential influence of a microbial entity on another microbial entity by incorporating information on the microbial abundances, because the abundances can limit the strength of the relevant metabolic influences via competitive or cooperative relationships between microbial entities. We start by quantifying the metabolic influence between two microbial species, as follows:

2. Two species interactions

Here, we estimate the potential metabolic influence of one species on another species. For a pair of species *i* and *j*, an increase in *i*'s abundance may contribute to an increase or decrease in *j*'s growth (in fact, we assume it to be *j*'s growth rate, or sometimes *j*'s abundance. Either assumption does not affect the following logic by much). *i*'s effect on *j*'s growth is expressed by $W_{ij} = \frac{(\partial\mu_j)/\mu_j}{(\partial n_i)/n_i}$, where μ_j is *j*'s growth rate, and n_i is *i*'s abundance. Both the numerator and the denominator adopt fold changes of their respective amounts. W_{ij}> 0 (< 0) indicates the positive (negative) metabolic influence that *i* is promotive (inhibitive) for *j*'s growth.

In more detail,

$$W_{ij} = \frac{(\partial\mu_j)/\mu_j}{(\partial n_i)/n_i} = \frac{n_i}{\mu_j}\cdot\left(\frac{\partial\mu_j}{\partial n_i}\right) = \frac{n_i}{\mu_j}\cdot\sum_k \frac{\partial\mu_j}{\partial z_{kj}}\frac{\partial z_{kj}}{\partial n_i}$$

where *k* denotes each metabolite imported by species *j*, and z_{kj} is the rate of metabolite *k* consumed by species *j* per unit abundance. Then, we proceed to calculate $\frac{\partial\mu_j}{\partial z_{kj}}$ and $\frac{\partial z_{kj}}{\partial n_i}$. For simplicity of analysis, we estimate $\mu_j = \bar{\Omega}_j \prod_l z_{lj}$, then $\frac{\partial\mu_j}{\partial z_{kj}} = \bar{\Omega}_j \prod_{l\neq k} z_{lj} = \frac{\mu_j}{z_{kj}}$. To calculate $\frac{\partial z_{kj}}{\partial n_i}$, we consider the following factors one-by-one:

2.1. Metabolic interactions between microbes

Under the steady-state condition, $\frac{dm_k}{dt} = 0$, where m_k is the concentration of metabolite *k* (this condition should be interpreted as a useful assumption to simplify our analysis, rather than taken strictly as is. As long as m_k has some characteristic value over time, the steady-state condition can be assumed as m_k's long-term behavior). Let P_k be the set of microbial species which produce metabolite *k*, C_k be the set of microbial species which consume metabolite *k*, S_{kx}^{P} be the rate of metabolite *k* produced by species *x* per unit abundance, S_{ky}^{C} be the rate of metabolite *k* consumed by species y per unit abundance, R_k^{in} be the influx of metabolite *k* into the gut environment, R_k^{out} be the outflux of metabolite *k* from the gut environment. Then,

$$\frac{dm_k}{dt} = \sum_{x\in P_k} S_{kx}^{\mathrm{P}} n_x - \sum_{y\in C_k} S_{ky}^{\mathrm{C}} n_y + R_k^{\mathrm{in}} - R_k^{\mathrm{out}} = 0$$

Although S_{kx}^{P} varies with *x*, we estimate $S_k^{\mathrm{P}} = \langle S_{kx}^{\mathrm{P}}\rangle_x$ for simplicity. Similarly, $S_k^{\mathrm{C}} = \langle S_{ky}^{\mathrm{C}}\rangle_y$. Let $R_k^{\mathrm{net}} \equiv R_k^{\mathrm{out}} - R_k^{\mathrm{in}}$, then

$$\frac{\mathrm{d}m_k}{dt} = S_k^{\mathrm{P}} \sum_{x\in P_k} n_x - S_k^{\mathrm{C}} \sum_{y\in C_k} n_y - R_k^{\mathrm{net}} = 0$$

Because $z_{kj} = S_k^{\mathrm{C}}$ (by definition),

$$z_{kj} = S_k^{\mathrm{C}} = (S_k^{\mathrm{P}} \sum_{x\in P_k} n_x - R_k^{\mathrm{net}}) / \sum_{y\in C_k} n_y$$

By taking the derivatives,

$$\frac{\partial z_{kj}}{\partial n_i} = \frac{S_k^{\mathrm{P}} - \Theta_{ki}}{\sum_y n_y} - \frac{S_k^{\mathrm{P}} \sum_x n_x - R_k^{\mathrm{net}}}{\left(\sum_y n_y\right)^2}$$

where $\Theta_{ki} \equiv \frac{\partial R_k^{\mathrm{net}}}{\partial n_i}$. Also by definition, we consider $\frac{S_k^{\mathrm{P}} - \Theta_{ki}}{\sum_y n_y}$ only when *i* is the producer of *k*, and we consider $\frac{S_k^{\mathrm{P}} \sum_x n_x - R_k^{\mathrm{net}}}{(\sum_y n_y)^2}$ only when *i* is the consumer of *k*. Hence,

$$W_{ij} = \frac{n_j}{\mu_i} \cdot \sum_k \frac{\partial \mu_j}{\partial z_{kj}} \frac{\partial z_{kj}}{\partial n_i} = \sum_k \left[\frac{n_i (1 - \Theta_{ki}/S_k^{\mathrm{P}})}{\sum_x n_x - R_k^{\mathrm{net}}/S_k^{\mathrm{P}}} - \frac{n_i}{\sum_y n_y} \right]$$

To simplify the above formula, we assume $R_k^{\mathrm{net}}/S_k^{\mathrm{P}} \ll \sum_x n_x$, then

$$W_{ij} \approx \sum_k \left[\frac{n_i}{\sum_x n_x} (1 - \Theta_{ki}/S_k^{\mathrm{P}}) \cdot (1 + \frac{R_k^{\mathrm{net}}}{S_k^{\mathrm{P}} \sum_x n_x}) - \frac{n_i}{\sum_y n_y} \right]$$

Let $\alpha = (1 - \Theta_{ki}/S_k^{\mathrm{P}}) \cdot (1 + \frac{R_k^{\mathrm{net}}}{S_k^{\mathrm{P}} \sum_x n_x})$,

$$W_{ij} \approx \sum_k \left[\frac{n_i}{\sum_x n_x} \alpha - \frac{n_i}{\sum_y n_y} \right]$$

where we estimate α as a constant, i.e., not varying much with respect to the identity of *k* and to species composition. An explanation of how we obtained the value for α is provided later.

Next, we introduce a threshold θ_1. If $\sum_{\mathrm{x}} n_x < \theta_1$ for a particular *k*, the first term inside the summation on the right-hand side ("producer term") is set to zero for this *k*. If $\sum_{\mathrm{y}} n_y < \theta_1$, the second term ("consumer term") is set to zero for this *k*. If $n_i < \theta_1$, all producer and consumer terms become zero regardless of *k*, and W_{ij} becomes zero. The reason why we introduce θ_1 is that, if producers or consumers of *k* are in very low abundance, they would not practically affect the growth of other species by *k*. Another reason is that, because *i*'s effect on the change of *j*'s abundance can be expressed as $\Delta n_j = W_{ij}(\frac{\Delta n_i}{n_i})$, we need to prevent a mathematical divergence when $n_i \to 0$ with a finite value W_{ij}.

2.2. Metabolic interactions between microbes and host cells

First, we consider the case where a host-produced metabolite *k* is consumed by a microbial species. Here, (*i*) metabolite *k* may be significantly produced by microbes, with $\sum_{\mathrm{x}} n_x \geq \theta_1$, or (*ii*) metabolite *k* may be mostly produced by the host, with $\sum_{\mathrm{x}} n_x < \theta_1$. For (*i*), we assume that *k* is produced more by microbes than by the host. Consequently, case (*i*) adds the terms $\frac{n_i}{\sum_x n_x} \alpha - \frac{n_i}{\sum_y n_y}$ to W_{ij}, while case (*ii*) adds the term $-\frac{n_i}{\sum_y n_y}$ to W_{ij}.

Second, we consider the case where both microbial species *j* and the host import metabolite *k*, and thus are in competition for *k*. In this case, the host would not affect the formula for the competition between microbial species, as they compete for the amount of metabolite *k* not consumed by the host, and as species *i*'s influence on species *j*'s growth is determined by how much species *i* takes this available metabolite *k* from species *j*. In other words, this case adopts the same formula for W_{ij} as (*i*) in the first case above.

Third, we consider the case where the host both produces and consumes metabolite *k*. This case considers the same situations (*i*) and (*ii*) described above, and thus adopts the same formula for W_{ij} from the respective situations.

2.3. Macromolecule degradation by microbes

To describe macromolecule degradation by microbes, we first consider a simple case where there is only one unique macromolecule which has metabolite *k* as one of its breakdown components. Next, we expand this case into those with multiple macromolecules having metabolite *k* as a common breakdown component. In the unique macromolecule case, under the steady-state condition of

metabolite *k*,

$$\frac{dm_k}{dt} = S_k^{\mathrm{P}_0} \sum_{x \in P_k^0} n_x + S_k^{\mathrm{P}_1} \prod_{z \in P_k^1} n_z - S_k^{\mathrm{C}} \sum_{y \in C_k} n_y - R_k^{\mathrm{net}} = 0$$

where P_k^0 is the set of species secreting metabolite *k*, P_k^1 is the set of species *z*'s degrading a particular macromolecule to release metabolite *k*. By assuming the synergistic effect when multiple species in P_k^1 work together to break down a macromolecule [*Appl. Environ. Microbiol.* **55**, 2247 (1989); *Microbiology* **140**, 3407 (1994)], we consider the multiplication, $S_k^{\mathrm{P}_1} \prod_{z \in P_k^1} n_z$, rather than the summation of individual species effects. In a similar approach described in Section 2.1, $\frac{\partial z_{kj}}{\partial n_i}$ is written as

$$\frac{\partial z_{kj}}{\partial n_i} = \frac{S_k^{\mathrm{P}_0} + S_k^{\mathrm{P}_1} \prod_{z \neq i} n_z - \Theta_{ki}}{\sum_y n_y} - \frac{S_k^{\mathrm{P}_0} \sum_x n_x + S_k^{\mathrm{P}_1} \prod_z n_z - R_k^{\mathrm{net}}}{\left(\sum_y n_y\right)^2}$$

where $S_k^{\mathrm{P}_0} + S_k^{\mathrm{P}_1} \prod_{z \neq i} n_z - \Theta_{ki}$ has $S_k^{\mathrm{P}_0}$ only when species *i* belongs to P_k^0; likewise, this term has $S_k^{\mathrm{P}_1} \prod_{z \neq i} n_z$ only when species *i* belongs to P_k^1. Consequently,

$$W_{ij} = \frac{n_j}{\mu_i} \cdot \sum_k \frac{\partial \mu_j}{\partial z_{kj}} \frac{\partial z_{kj}}{\partial n_i} = \sum_k \left[\frac{n_i \left(S_k^{\mathrm{P}_0} + S_k^{\mathrm{P}_1} \prod_{z \neq i} n_z - \Theta_{ki} \right)}{S_k^{\mathrm{P}_0} \sum_x n_x + S_k^{\mathrm{P}_1} \prod_z n_z - R_k^{\mathrm{net}}} - \frac{n_i}{\sum_y n_y} \right]$$

where every term in the denominator $S_k^{\mathrm{P}_0} \sum_x n_x + S_k^{\mathrm{P}_1} \prod_z n_z - R_k^{\mathrm{net}}$ is always present regardless of whether *i* belongs to P_k^0 or not, and whether *i* belongs to P_k^1 or not. Only the terms in the numerator $S_k^{\mathrm{P}_0} + S_k^{\mathrm{P}_1} \prod_{z \neq i} n_z - \Theta_{ki}$ depend on *i*'s membership, as described above. Here, $\frac{n_i \left(S_k^{\mathrm{P}_0} + S_k^{\mathrm{P}_1} \prod_{z \neq i} n_z - \Theta_{ki} \right)}{S_k^{\mathrm{P}_0} \sum_x n_x + S_k^{\mathrm{P}_1} \prod_z n_z - R_k^{\mathrm{net}}}$ corresponds to the producer term, and $\frac{n_i}{\sum_y n_y}$ corresponds to the consumer term. We further assume $S_k^{\mathrm{P}_1} \gg S_k^{\mathrm{P}_0}$, indicating that the production rate of metabolite *k* by macromolecule breakdown is generally much higher than that by microbial export. For example, both macromolecule degradation (such as proteolysis) and microbial export can provide amino acids, yet, the former reflects an active foraging process, while the latter is likely to be a rarer event that involves temporal dysregulation of amino acid metabolism [*J. Appl. Bacteriol.* **64**, 37 (1988); *Nat. Rev. Microbiol.* **12**, 327 (2014)]. If $\sum_z n_z < \theta_1$, one can take the same approach shown in Section 2.1. But, if $\sum_z n_z \geq \theta_1$ and *j* does not belong to P_k^1, the condition $S_k^{\mathrm{P}_1} \gg S_k^{\mathrm{P}_0}$ leads to a zero producer term for *k* in W_{ij}. If $\sum_z n_z \geq \theta_1$ and *i* belongs to P_k^1, the condition $S_k^{\mathrm{P}_1} \gg S_k^{\mathrm{P}_0}$ leads to the producer term of $(1 - \Theta_{ki}/(S_k^{\mathrm{P}_1} \prod_{z \neq i} n_z))/(1 - R_k^{\mathrm{net}}/(S_k^{\mathrm{P}_1} \prod_z n_z))$. In this case, (*i*) if species *i*'s abundance is large enough with $n_i \geq \theta_1$, we estimate the producer term to be some constant, β. (*ii*) If species *i*'s abundance is small with $n_i < \theta_1$, we assume that the producer term becomes smaller and eventually zero as $n_i \to 0$, and set the producer term to be $\left(\frac{n_i}{\sum_z n_z}\right)\beta$. β is estimated to be a constant that does not vary much with the identities of macromolecules and breakdown products.

We consider the multiple macromolecule case when metabolite *k* is a breakdown product of macromolecule 1, 2, ⋯, *q*. P_k^m is defined as a set of species that degrade macromolecule *m* and release metabolite *k*. Then,

$$W_{ij} = \frac{n_j}{\mu_i} \cdot \sum_k \frac{\partial \mu_j}{\partial z_{kj}} \frac{\partial z_{kj}}{\partial n_i} = \sum_k \left[\frac{S_k^{\mathrm{P}_0} n_i + \sum_{P_k^m \ni i} S_k^{\mathrm{P}_m} \prod_{z \in P_k^m} n_z - \Theta_{ki} n_i}{S_k^{\mathrm{P}_0} \sum_x n_x + \sum_{P_k^m} S_k^{\mathrm{P}_m} \prod_{z \in P_k^m} n_z - R_k^{\mathrm{net}}} - \frac{n_i}{\sum_y n_y} \right]$$

We further assume that $S_k^{\mathrm{P}_m}$ does not have strong *m*-dependency (given the scarcity of quantitative information on $S_k^{\mathrm{P}_m}$, this is the simplest assumption that we can make for our analysis), and we define *N* and *n* as follows: *N* is the number of macromolecule *m*'s satisfying $\sum_{z \in P_k^m} n_z \geq \theta_1$, and *n* is the number of macromolecule *m*'s which are degraded by species *i* and satisfy $\sum_{z \in P_k^m} n_z \geq \theta_1$. If *N* > 0,

the producer term is

$$\frac{n}{N}\beta \;\; \text{if}\, n_i \geq \theta_1$$

or

$$\frac{\beta}{N}\sum_{PP_k^m}\frac{n_i}{\sum_{z\in P_k^m} n_z} \;\; \text{if}\, n_i < \theta_1$$

where the second formula's PP_k^m denotes the index of macromolecule *m* which is degraded by species *i* and satisfies $\sum_{z\in P_k^m} n_z \geq \theta_1$. On the other hand, if *N* = 0, we follow the same approach as in Section 2.1, because no macromolecule would be effectively degraded in this case.

2.4. Metabolic compounds from dietary or abundant endogenous sources

Among all metabolites considered for interspecies cross-feeding, some may have dietary sources or unspecified intestinal sources (e.g., sloughed epithelial cells and secreted proteins). If a given metabolite corresponds to the latter case and has a large abundance in the intestine, we exclude this metabolite when calculating W_{ij}. The reason is that this abundant metabolite production would not depend significantly on particular species or particular host components, and thus would not be a limiting factor in metabolic interactions between microbes, and between microbes and the host. In the case where a given metabolite is a small molecule and is from dietary sources (but not unspecified endogenous sources), the availability of this small molecule (if the molecule is produced by microbes as well) would be continuous in time by constant microbial production rather than by dietary supply that would be shortly exhausted. In other words, this metabolite still plays a significant role in interspecies metabolic interactions. If a given metabolite is a constituent of diet-derived macromolecules, one can follow a similar scheme to Section 2.3. Taken together, if the number of species which export (dietary) small molecule *k* is ≥ θ_1, or if the number of species which degrades (dietary) macromolecules to release metabolite *k* is ≥ θ_1, we follow the same schemes as Sections 2.1 to 2.3. Otherwise, we discard such dietary compounds as they may hardly affect interspecies metabolic interactions. Of note, when n_i=0, W_{ij}=0.

3. Microbial group-to-group interactions

Thus far, we have considered metabolic influences involving individual microbial species. Because our microbial entities do not only comprise individual species, but also groups of species such as genera and metabolic cliques, we here quantify metabolic influences involving those groups. First, we consider a simple case where a group exerts a metabolic influence upon a single species. For species *i* and group *G*, we check how a change in group *G*'s abundance affects species *i*'s growth, i.e., how individual species in *G* collectively affect *i*'s growth. Since any given *G* is composed of multiple species,

$$W_{Gi} = \frac{(\partial\mu_i)/\mu_i}{(\partial n_G)/n_G} = \frac{n_G}{\mu_i}\sum_{l\in G}\frac{\partial\mu_i}{\partial n_l}/\frac{dn_G}{dn_l}$$

Based on the lowest-order approximation, we assume that the proportion of each species inside *G* stays almost constant when the total abundance of *G* becomes changed. In other words, for the abundance (n_l) of species *i* in *G*, $\frac{dn_G}{dn_l} = \frac{n_G}{n_l}$. Therefore,

$$W_{Gi} = \frac{n_G}{\mu_i}\sum_{l\in G}\frac{\partial\mu_i}{\partial n_l}/\frac{dn_G}{dn_l} = \frac{n_G}{\mu_i}\sum_{l\in G}\frac{\partial\mu_i}{\partial n_l}\cdot\frac{n_l}{n_G} = \sum_{l\in G}\frac{(\partial\mu_i)/\mu_i}{(\partial n_l)/n_l} = \sum_{l\in G} W_{li}$$

Consequently, the metabolic influence of *G* to *i* is equivalent to the summation of the individual group members' metabolic influences.

Second, we consider the metabolic influence of species *i* upon group *G* as,

$$W_{iG} = \frac{(\partial\mu_G)/\mu_G}{(\partial n_i)/n_i}$$

Because μ_G is positively correlated with the total abundance of its group members, we estimate $\mu_G \approx \sum_{l \in G} n_l$. Then, $\partial\mu_G \approx \sum_{l \in G} \partial n_l$, and

$$W_{iG} = \frac{(\partial\mu_G)/\mu_G}{(\partial n_i)/n_i} \approx \frac{n_\mathrm{i}}{\mu_G}\sum_{l \in G}\frac{\partial n_l}{\partial n_i} = \sum_{l \in G}\frac{n_l}{\mu_G}\frac{\partial n_l/n_l}{\partial n_i/n_i} = \sum_{l \in G}\frac{n_l}{\mu_G}W_{il}$$

Therefore, the metabolic influence is the weighted sum over the species members in *G*, and each weight is proportional to each species' abundance. Lastly, we consider the metabolic influence of a group upon another group. For group *G* and group *Γ*, which do not share any species in common, we calculate the metabolic influence of *G* upon *Γ*. For *G*, we again assume that each species proportion is almost kept constant.

$$W_{G\Gamma} = \frac{(\partial\mu_\Gamma)/\mu_\Gamma}{(\partial n_G)/n_G} \approx \frac{n_G}{\mu_\Gamma}\sum_{l \in G}\frac{\partial\mu_\Gamma}{\partial n_l}\cdot\frac{dn_l}{dn_G} = \frac{n_G}{\mu_\Gamma}\sum_{l \in G}\frac{\partial\mu_\Gamma}{\partial n_l}\cdot\frac{n_l}{n_G} = \frac{1}{\mu_\Gamma}\sum_{l \in G}\frac{\partial\mu_\Gamma}{\partial n_l/n_l}$$

$$\approx \frac{1}{\mu_\Gamma}\sum_{l \in G}\sum_{q \in \Gamma}\frac{\partial\mu_q}{\partial n_l/n_l} = \sum_{l \in G}\sum_{q \in \Gamma}\frac{\mu_q}{\mu_\Gamma}\left(\frac{\partial\mu_q/\mu_q}{\partial n_l/n_l}\right) = \sum_{l \in G}\sum_{q \in \Gamma}\frac{\mu_q}{\mu_\Gamma}W_{lq}$$

Therefore, the metabolic influence includes the weighted sum over member species in *Γ*, and each weight is proportional to each species' abundance. If n_Γ = 0, we set $\frac{\mu_q}{\mu_\Gamma} = 1/(\#\text{ of microbial species in }\Gamma)$, because every species in *Γ* has the same zero abundance. Importantly, when groups *G* and *Γ* share common species, we calculate the metabolic influence of *G* upon *Γ* by using *Γ−G,* instead of simply *Γ*. This substitution prevents a redundant metabolic influence of *G* upon itself, as evident in the case that *G* is a subset of *Γ*.

4. Regarding microbial abundance

For species *i*, n_i and n_i+Δn_i in Sections 1 to 3 are the median value of *i*'s relative abundances over all control samples and that over all T2D samples, respectively (the latter is not entirely true, though, as will be explained later). This usage is motivated by observations that a control state can undergo a transformation into a T2D state, but the reverse is less likely. In other words, n_i in W_{ij} corresponds to the microbial abundance in control samples.

5. Consistency between metabolic influences and abundance changes

We assign a directed link with weight W_{ij}, from one microbial entity *i* to another entity *j*, and vice versa. For every pair of differentially abundant or scarce microbial entities in T2D, we assign those links. Among those links, we consider only the links that are consistent with actual microbial abundance changes between control and T2D subjects. For this consistency check, we introduce the notations for link types: + (W>0), − (W<0), and x (no link). In the order of directed links *i→j* and *j→i*, we write the two link types; for example, +− indicates that *i→j* is + and *j→i* is −. In the case with xx, *i* and *j* are fully disconnected, so we do not examine its trivial, consistency issue. For the other two cases, the consistency condition can be met if the following conditions are satisfied:

(*i*) ++ : both microbial entities should exhibit abundance changes in the same directions, i.e., either both differentially abundant or both differentially scarce in T2D.

(*ii*) −− : the two microbial entities should exhibit abundance changes in the opposite directions, i.e., one being differentially abundant and the other being differentially scarce in T2D.

(*iii*) +− : this case is more complex than the previous two cases. Let $n_i(0)$ and $n_j(0)$ be entity *i*'s and entity *j*'s initial abundances, respectively. Then, the entity *j*'s abundance change Δn_j made by the entity *i*'s abundance change Δn_i is given by the following formula, from $W_{ij} = \frac{(\partial\mu_j)/\mu_j}{(\partial n_i)/n_i}$:

$$\Delta\mu_j = W_{ij}\frac{\mu_j}{n_i}\Delta n_i$$

where μ_j is estimated to be *j*'s abundance. Likewise,

$$\Delta\mu_i = W_{ji}\frac{\mu_i}{n_j}\Delta n_j$$

In an iterative manner over time (*t*), the abundance of one entity will change according to the abundance of the other, as follows:

t	$n_i(t)$	$n_j(t)$
0	$n_i(0)$	$n_j(0)$
1	$n_i(0) + \Delta n_i$	$n_j(0) + \Delta n_j$
2	$n_i(1) + W_{ji}\frac{\mu_i(1)}{n_j(0)}\Delta n_j$	$n_j(1) + W_{ij}\frac{\mu_j(1)}{n_i(0)}\Delta n_i$
3	$n_i(2) + W_{ji}\frac{\mu_i(2)}{n_j(1)}\Delta n_j(2)$	$n_j(2) + W_{ij}\frac{\mu_j(2)}{n_i(1)}\Delta n_i(2)$
4	$n_i(3) + W_{ji}\frac{\mu_i(3)}{n_j(2)}\Delta n_j(3)$	$n_j(3) + W_{ij}\frac{\mu_j(3)}{n_i(2)}\Delta n_i(3)$

where $\Delta n_i(t) = n_i(t) - n_i(t-1)$ and $\Delta n_j(t) = n_j(t) - n_j(t-1)$. Generally,

$$\Delta n_j(t) = W_{ij}\frac{\mu_j(t-1)}{n_i(t-2)}\Delta n_i(t-1)$$

and the same formula is held for Δn_i by exchanging *i* and *j*.

Next, we examine the asymptotic behavior when $t \rightarrow \infty$. We start with the following equations:

$$\frac{\Delta n_i(t)}{n_i(t-1)} = W_{ji}\frac{\Delta n_j(t-1)}{n_j(t-2)}$$

$$\frac{\Delta n_j(t)}{n_j(t-1)} = W_{ij}\frac{\Delta n_i(t-1)}{n_i(t-2)}$$

They lead to

$$\frac{\Delta n_i(2t+1)}{n_i(2t)} = W_{ij}W_{ji}\frac{\Delta n_i(2t-1)}{n_i(2t-2)}$$

Let $\overline{w}_{ij} \equiv W_{ij}W_{ji}$, then,

$$\frac{\Delta n_i(2t+1)}{n_i(2t)} = (\overline{w}_{ij})^t\frac{\Delta n_i}{n_i(0)}$$

Because $\Delta n_i(2t+1) = n_i(2t+1) - n_i(2t)$,

$$n_i(2t+1) = \left[1 + (\overline{w}_{ij})^t\frac{\Delta n_i}{n_i(0)}\right]n_i(2t)$$

Similarly,

$$n_i(2t) = \left[1 + (\overline{w}_{ij})^{t-1}W_{ji}\frac{\Delta n_j}{n_j(0)}\right]n_i(2t-1)$$

Consequently,

$$n_i(2t) = n_i(0)\prod_{k=0}^{t-1}\left[1 + (\overline{w}_{ij})^k W_{ji}\frac{\Delta n_j}{n_j(0)}\right]\left[1 + (\overline{w}_{ij})^k\frac{\Delta n_i}{n_i(0)}\right]$$

$$n_i(2t+1) = n_i(0)\left[1 + (\overline{w}_{ij})^t\frac{\Delta n_i}{n_i(0)}\right]\prod_{k=0}^{t-1}\left[1 + (\overline{w}_{ij})^k W_{ji}\frac{\Delta n_j}{n_j(0)}\right]\left[1 + (\overline{w}_{ij})^k\frac{\Delta n_i}{n_i(0)}\right]$$

If $|\overline{w}_{ij}| \ll 1$, then $n_i(\infty)$ converges; else, it diverges with oscillation over time. Therefore, we only consider the cases with $|\overline{w}_{ij}| \ll 1$ for further analyses; otherwise, we simply do not assign a link between entities *i* and *j*. Specifically, we consider the cases with $|\overline{w}_{ij}| < \theta_2$, wherein θ_2 will be determined later.

When $|\bar{w}_{ij}| \ll 1$, the first-order approximation with $\bar{w}_{ij}$, Δn_i, and Δn_j gives rise to

$$n_i(\infty) \approx n_i(0) + [1 + \bar{w}_{ij}]\Delta n_i + [1 + \bar{w}_{ij}]W_{ji}\frac{n_i(0)}{n_j(0)}\Delta n_j$$

Similarly,

$$n_j(\infty) \approx n_j(0) + [1 + \bar{w}_{ij}]\Delta n_j + [1 + \bar{w}_{ij}]W_{ij}\frac{n_j(0)}{n_i(0)}\Delta n_i$$

Let $\Delta n_i^f \equiv n_i(\infty) - n_i(0)$ and $\Delta n_j^f \equiv n_j(\infty) - n_j(0)$, then

$$[1 + \bar{w}_{ij}]\begin{bmatrix} 1 & W_{ji}\dfrac{n_i(0)}{n_j(0)} \\ W_{ij}\dfrac{n_j(0)}{n_i(0)} & 1 \end{bmatrix}\begin{bmatrix}\Delta n_i \\ \Delta n_j\end{bmatrix} = \begin{bmatrix}\Delta n_i^f \\ \Delta n_j^f\end{bmatrix}$$

Therefore,

$$\begin{bmatrix}\Delta n_i \\ \Delta n_j\end{bmatrix} = \frac{1}{1 - \bar{w}_{ij}^2}\begin{bmatrix} \Delta n_i^f - W_{ji}\dfrac{n_i(0)}{n_j(0)}\Delta n_j^f \\ \Delta n_j^f - W_{ij}\dfrac{n_j(0)}{n_i(0)}\Delta n_i^f \end{bmatrix}$$

Here, $n_{i(j)}(0)$ and $\Delta n_{i(j)}^f$ can be obtained from microbial abundance data from T2D and control subjects. Because a control state can undergo a transformation into a T2D state, but the reverse is less likely, $n_i(0)$ corresponds to the abundance of species *i* in control subjects, and $n_i(\infty)$ corresponds to that in T2D subjects. Using this information of $n_i(0)$ and $n_i(\infty)$ (or Δn_i^f), the above equation gives the values of Δn_i and Δn_j. If the resulting Δn_i and Δn_j are within a reasonable range, the consistency condition can be regarded as satisfied. Otherwise, it cannot. Hence, we need to determine what would be the reasonable range of Δn_i and Δn_j, as follows:

$$0 \le n_i(1) \le n_i^{\max}$$
$$0 \le n_j(1) \le n_j^{\max}$$

The above inequalities can be converted into

$$-n_i(0) \le \Delta n_i \le n_i^{\max} - n_i(0)$$
$$-n_j(0) \le \Delta n_j \le n_j^{\max} - n_j(0)$$

When the values of Δn_i and Δn_j satisfy these two inequalities simultaneously, the consistency condition is satisfied. If they do not for either of the inequalities, the consistency condition is not satisfied. The method to obtain $n_i^{\max}$ will be explained later.

In the case where both n_i and n_j are not zero but W_{ji}=0, the exact solution leads to (*iv*) below. Likewise, in the case where $n_{i(j)}(0)$=0 and thus $W_{ij(ji)}$=0, refer to (*iv*) below.

(*iv*) +x (or –x): suppose that, at the initial time, entity *i*'s abundance $n_i(0)$ is changed by Δn_i and entity *j*'s abundance $n_j(0)$ is changed by Δn_j. Because $W_{ji} = 0$, Δn_i affects entity *j*'s abundance only once, as follows:

t	$n_i(t)$	$n_j(t)$
0	$n_i(0)$	$n_j(0)$
1	$n_i(0) + \Delta n_i$	$n_j(0) + \Delta n_j$
2	$n_i(0) + \Delta n_i$	$n_j(1) + W_{ij}\dfrac{\mu_j(1)}{n_i(0)}\Delta n_i$

Therefore, $n_i(0) + \Delta n_i = n_i(\infty)$ and $\Delta n_i = \Delta n_i^f$. On the other hand,

$$n_j(1) + W_{ij}\frac{\mu_j(1)}{n_i(0)}\Delta n_i = n_j(\infty)$$

Combined with $\mu_j(1) \approx n_j(1)$, this leads to

$$n_j(1) = n_j(\infty)/\left[1 + W_{ij}\frac{\Delta n_i^f}{n_i(0)}\right]$$

Since $n_j(1) = n_j(0) + \Delta n_j$, then

$$\Delta n_j = \left[\Delta n_j^f - W_{ij}\frac{n_j(0)}{n_i(0)}\Delta n_i^f\right]/\left[1 + W_{ij}\frac{\Delta n_i^f}{n_i(0)}\right]$$

This entity pair meets the consistency condition only when satisfying the following three inequalities:

$$0 \le n_i(1) \le n_i^{\max}$$
$$0 \le n_j(1) \le n_j^{\max}$$
$$0 \le n_j(\infty) \le n_i^{\max}$$

Let $\gamma = \left[1 + W_{ij}\frac{\Delta n_i^f}{n_i(0)}\right]$, then the above inequalities are equivalent to

$$0 \le \gamma$$
$$0 \le n_i(\infty) \le n_i^{\max}$$
$$\begin{cases} 0 \le n_j(\infty) \le \gamma n_j^{\max} & \text{if } 0 \le \gamma < 1 \\ 0 \le n_j(\infty) \le n_j^{\max} & \text{if } 1 < \gamma \end{cases}$$

In the case with $\gamma = 0$, $n_j(2) = 0$ regardless of $n_j(0)$. Therefore, $n_j(\infty)$ = 0 regardless of Δn_j. It means that, as long as $n_j(\infty)$ = 0, the consistency condition is satisfied if the condition for the entity *i* is satisfied.

(*v*) Additional link filtering: for pairs of microbial entities pertaining to (*iii*) and (*iv*), we perform additional link filtering. When link *i*→*j* is + and link *j*→*i* is −, and when *i* and *j* are differentially abundant in T2D, we remove link *j*→*i*. This is because the putative metabolic influence *j*→*i* can not clearly account for entity *i*'s elevation in T2D; there can be more plausible causes of entity *i*'s elevation in T2D, such as metabolic influence *i*→*j*, or other (non-metabolic) factors which are beyond our study's scope [*Infect. Immun.* **73**, 3197 (2005); *Proc. Natl. Acad. Sci. USA* **113**, 3639 (2016)]. These filtering schemes for (*iii*) and (*iv*) are summarized in the table below (up, differentially abundant; down, differentially scarce; O, not removed; X, removed):

Link types	Abundance change in T2D (*i* / *j*)	*i*→*j*	*j*→*i*
+−	up/up	O	X
	up/down	X	O
	down/up	X	O
	down/down	O	X
+x	up/up	O	X
	up/down	X	X
	down/up	X	X
	down/down	O	X
−x	up/up	X	X
	up/down	O	X
	down/up	O	X
	down/down	X	X

6. Parameter estimation

(*i*) θ_1: by inspecting species abundance distributions, we adopt θ_1= 0.001%.
(*ii*) The consistency of each link depends on the values of *α* and *β*. We take the values of *α* and *β*

that relatively well satisfy the consistency conditions. To find α and β, we start by considering microbial entities which passed the redundancy test for differential abundance. The following two cases have clear consistency conditions: ++ and −−. The former satisfies the consistency condition when two microbial entities are both differentially abundant or both differentially scarce, while the latter does when one entity is differentially abundant and the other entity is differentially scarce. We index a pair of entities in two ways: First, ++ or −− (the other cases like +− are discarded in this consistency analysis). Second, A or B. If two entities in a pair are both differentially abundant or both differentially scarce, A. If one entity is differentially abundant and the other entity is differentially scarce, B (the other cases are discarded in this consistency analysis). Then, we count the number of pairs corresponding to each of these 2^2 cases. For example, n_{A}^{++} denotes the number of pairs, each of which has both differentially abundant or both differentially scarce entities, with positive metabolic influences towards one another. The consistency score is given by

$$\frac{n_{\mathrm{A}}^{++}+n_{\mathrm{B}}^{--}}{n_{\mathrm{A}}^{++}+n_{\mathrm{A}}^{--}+n_{\mathrm{B}}^{++}+n_{\mathrm{B}}^{--}}$$

We search for α and β that give relatively favorable consistency scores. As a result, we adopt α = 1 and β = 0.1.

(*iii*) The value of θ_2 is determined by inspecting a distribution of $\overline{\mathrm{w}}_{ij}<0$ with the values of α and β obtained in (*ii*). We adopt $\theta_2=0.1$.

(*iv*) To determine n_i^{max}, we first obtain $n_i(0)$ (median abundance from control samples) and $n_i(\infty)$ (median abundance from T2D samples) for microbial entity *i*. Let A_i be a set of all $n_i(0)$'s and $n_i(\infty)$'s, m_i be the average of all elements in A_i, and s_i be the standard deviation of all elements in A_i. We further define

$$f_i=\begin{cases}\text{largest } n_i \text{ in } A_i \text{ if } m_i+2s_i>1\\ \max(\text{largest } n_i \text{ in } A_i, m_i+2s_i), \text{otherwise}\end{cases}$$

The idea behind f_i is to assume that n_i^{max} roughly corresponds to a value with a z-score of 2. By adding some buffer to f_i, we estimate n_i^{max} to be 1.1f_i, but no more than 1, as $n_i^{max}=\max(1,1.1f_i)$.

7. Microbe-host interactions

We identify specific microbe-host interactions that are representative of a given cohort. Along the same line of reasoning mentioned above, in this procedure we do not consider metabolites in large abundance with unspecified intestinal sources (e.g., sloughed epithelial cells and secreted proteins), because these metabolites would not be limiting factors in metabolic interactions between microbes and the host. In the case with metabolites from dietary sources (but not from unspecified endogenous sources), if the abundance of species which export (dietary) small molecule *k* is ≥ θ_1, or if the abundance of species which degrade (dietary) macromolecules containing metabolite *k* is ≥ θ_1, we consider this metabolite *k* in our analysis. Otherwise, we discard such dietary compounds, as they may hardly affect microbe-host metabolic interactions.

7.1. Microbial influence on the host

Microbes can exert a metabolic influence on the host by providing (for host consumption) small molecule *k*, via either metabolite secretion or macromolecule breakdown. If metabolite *k*'s overall production is differentiated between T2D and control, that metabolite may play a role in the context of the host phenotype (T2D or control). For each metabolite *k* consumed by the host (among those satisfying the aforementioned conditions, such as without abundant unspecified intestinal sources), we screen metabolic cliques that export (solely export or both export and import) metabolite *k* or release this metabolite by macromolecule degradation. Note that these metabolic cliques are among those cliques before being filtered out by the redundancy test for differential abundance. If these

multiple metabolic cliques to produce metabolite *k* are altogether differentially abundant or scarce in T2D, we consider metabolite *k* in our analyses; otherwise, we discard it from further analyses. For the resulting metabolite *k*, if the corresponding metabolic cliques are differentially abundant (scarce) in T2D, we choose microbial entities that are also differentially abundant (scarce) in T2D, among those which pass the redundancy test for differential abundance, and produce metabolite *k* through secretion or macromolecule degradation.

7.2. Host influence on microbes

The host cell can exert a metabolic influence on microbial cells through its own produced compounds, i.e., small molecules or macromolecules that are utilized by microbes. Among the host-derived compounds (satisfying the aforementioned conditions, such as without abundant unspecified intestinal sources), we exclude metabolites that are also produced by microbes, as we assume that production of these metabolites is attributed more to the microbes than to the host. For a given compound, we screen for metabolic cliques that import (solely import or both import and export) or degrade that compound. Note that these metabolic cliques are among those cliques before being filtered out by the redundancy test for differential abundance. If these multiple metabolic cliques are altogether differentially abundant or scarce in T2D, we consider this compound in our analyses; otherwise, we discard it from further analyses. For the resulting compound, if the corresponding metabolic cliques are differentially abundant (scarce) in T2D, we choose microbial entities which are also differentially abundant (scarce) in T2D, among those which passed the redundancy test for differential abundance and import or degrade that compound.

8. Identification of influencers

Thus far, we have presented the method to construct a microbial metabolic influence network. In this network, we can identify community influencers, which are microbial entities that exert a very positive or negative metabolic influence on many other microbial entities in the network. Here, we only consider metabolic influences which are relevant to the host phenotype difference, T2D versus control.

Given microbial entities *i* and *j*, entity *i* can have a metabolic influence on entity *j* if they are directly connected to each other in the influence network. Even when they do not have a direct connection, entity *i* may eventually exert a metabolic influence on entity *j*, if other entities are located between entities *i* and *j* and thereby indirectly transfer entity *i*'s influence to entity *j*. For entities *i* and *j*,

$$\frac{\Delta n_j}{n_j} = \sum_k W_{kj} \frac{\Delta n_k}{n_k} = \frac{\Delta n_i}{n_i} \sum_P \prod_{l,m \in P} W_{lm}$$

where *P* denotes a path from *i* to *j*, and *l* and *m* are any entities consecutively linked along *P*. What if $|W_{lm}|$ exceeds 1? In this case, an entity farther from *j* exerts a stronger effect (on *j*) than *l* and *m*. However, we assume that the closer an entity is to *j*, the stronger its influence is on *j*. Mathematically, for any entity *k* (except *i* and *j*) along a path *P*, it should be satisfied that $|\Phi_{ij}^P| \leq |\Phi_{kj}^P|$ and $|\Phi_{ij}^P| \leq |\Phi_{ik}^P|$, where Φ_{ij}^P is the degree of metabolic influence from *i* to *j* through *P*. The first condition leads us to compare $\Phi_{kj}^P = W_{j-1,j} W_{j-2,j-1} \cdots W_{k,k+1} \Delta n_k / n_k$ and $\Phi_{k-1,j}^P = W_{j-1,j} W_{j-2,j-1} \cdots W_{k,k+1} W_{k-1,k} \Delta n_{k-1} / n_{k-1}$ and ensures that the latter has a magnitude no greater than the former. In other words, $\left|\frac{\Delta n_k}{n_k}\right| \geq \left|\frac{W_{k-1,k} \Delta n_{k-1}}{n_{k-1}}\right|$, and thus $\left|\frac{\Delta n_k n_{k-1}}{n_k \Delta n_{k-1}}\right| \geq |W_{k-1,k}|$ for every *k* except *j*. The second condition requires, for every *k* except *i*, $|W_{k,k+1}| \leq 1$. To summarize, we calculate Φ_{ij}^P by transforming $W_{k,k+1}$ into

$$|W_{k,k+1}| = \begin{cases} \min\left(|W_{k,k+1}|, \left|\frac{\Delta n_{k+1} n_k}{n_{k+1} \Delta n_k}\right|\right) \text{for } k = i \\ \min\left(|W_{k,k+1}|, \left|\frac{\Delta n_{k+1} n_k}{n_{k+1} \Delta n_k}\right|, 1\right) \text{for } i < k < j \\ \min(|W_{k,k+1}|, 1) \text{ for } k = j \end{cases}$$

In this transformation, we keep the sign of $W_{k,k+1}$ as is. We calculate the above by counting *k* away from *j*. If one encounters $\Delta n_k = 0$, we treat $\left|\frac{\Delta n_{k+1} n_k}{n_{k+1} \Delta n_k}\right|$ as the infinite, which in turn results in $W_{k+2,k+1} = 0$. Consequently, we obtain Φ_{ij}^{P} for a single path *P*. If multiple paths exist between the entities *i* and *j*, we consider the shortest paths among them. One may quantify *i*'s influence on *j* (Φ_{ij}) either as the sum or the average of Φ_{ij}^{P} over those shortest path *P*'s. Because the latter is supposed to be more robust to noises in the data (although the former is conceptually more correct), we chose the latter and calculated Φ_{ij} accordingly. Multiplying this Φ_{ij} by $\mathrm{sgn}(\Delta n_i)$ gives rise to the final value of Φ_{ij}.

Next, we quantify the overall influence of entity *i* throughout the entire influence network (Φ_i). For each entity *i*, Φ_i is defined as the number of entity *j*'s that satisfy $|\Phi_{ij}| \geq \theta_\Phi$ ($\theta_\Phi = 0.001$). Here, the value of θ_Φ was determined to reveal the full heterogeneity of Φ_i over every entity *i*, by observing the distribution of Φ_i across different values of θ_Φ. Lastly, we identify community influencers, which are microbial entities with distinctively large Φ_i's. Specifically, we identify a transition point of Φ_i from the probability distribution of Φ_i (Fig. 5a), which distinguishes one group of microbial entities (shaded area in Fig. 5a) from the other in their Φ_i's, and we use this transition point of Φ_i as the lower bound of the influencers' Φ_i.

9. Practical summary

The aforementioned procedures can be implemented as follows:

(*i*) To calculate W_{ij} for each link, first obtain α and β as shown in Section 6.

(*ii*) Using α and β, calculate W_{ij} for a link between species, between groups, and between a species and a group. If $|W_{ij}|$ is too small, the sign of W_{ij} would be susceptible to noise, which could cause uncertainty in the consistency for a given entity pair (see Section 5). Therefore, we remove links with $|W_{ij}| < \theta_3$ (the distribution of $|W_{ij}|$ suggests $\theta_3 = 10^{-4}$).

(*iii*) Using α, β, and W_{ij}, calculate θ_2 and $n_i^{\max}$ as shown in Section 6.

(*iv*) Using W_{ij}, check the consistency condition in Section 5 for each pair of differentially abundant or scarce entities. Remove links that do not satisfy the consistency condition.

(*v*) Among the links of +–, +x, and –x, perform the additional filtering described in Section 5.

(*vi*) Follow Section 7. In this step for microbe-host interactions, do not discard microbial entities that become isolated after link filtering in previous steps.

(*vii*) Follow Section 8.
