# Supplementary material for: Global metabolic interaction network of the human gut microbiota for context-specific community-scale analysis
Source: Nat Commun. 2017 Jun 6;8:15393. doi: 10.1038/ncomms15393 (PMC5467172; doi:10.1038/ncomms15393)
Supplement: Supplementary Software — Codes for the computation of microbe-microbe and microbe-host metabolic influences. [file ncomms15393-s6.zip › Microbe_microbe_influence/README.pdf]

README.pdf: Updated on 01/08/2017

This directory contains sample data to identify metabolic influence (W_{ij})s between all ordered pairs of microbial entities. Annotations of the numerical IDs representing microbial species, groups, and chemical compounds (used in the sample data) are provided in **annotations.xlsx**.

All input files are in the "Input_files" directory and all output files will be created in the "Output_files" directory upon successful completion of running the program. The names of the input files are fixed and written in the code; different input file name(s) will create errors while running the program. The final output is the following three files:

- met_microbe_coupling.txt
- node_node_ABC.txt
- Wmatrix_a1.0_b1.0_thre1e-4.txt

The last file is the global metabolic influence matrix (W-matrix). This W-matrix is an *n* x *n* matrix, wherein *n* is the total number of microbial entities (microbial species and groups). A value in the $(i,j)^{th}$ position of the matrix can be interpreted as the microbial influence (W_{ij}) of entity *i* (of row *i*) towards entity *j* (of column *j*). The top-to-bottom row order, as well as the left-to-right column order, of the microbial entities goes by the top-to-bottom order of the microbial species provided in **microbe_info.txt**, followed by the top-to-bottom order of microbial groups provided in **group_info.txt** (see **annotations.xlsx**).

For Windows users:

We used Microsoft Visual Studio 2010. If you open "Calculating_W_matrix.sln" and run the program, then you can obtain the results.

For Linux users:

Within the terminal, move the current directory to the "Linux_code" directory. If you type "make" (excluding quotation marks), then you can obtain the program file "getWmatrix". Use "make clean" to remove the object files. The standard C++ 11 should be supported to build the final program.

Definition of Input Files

1. Information on *relative abundances of microbial species*

File name: **microbe_info.txt**

Format: First row shows the total number of microbial species in the microbial metabolic transport network. From the second row and onward, each row is in the format of (tab-delimited): (species ID) (median abundance in healthy samples) (median abundance in T2D samples) (differentially abundant? 1: if in control; -1: if in T2D; 0: not differentially abundant).

2. List of *microbial group members*

File name: **group_info.txt**

Format: First row shows the total number of groups, i.e. genera and/or metabolic cliques. From the second row and onward, each row is in the format of (tab-delimited): (group ID) (differentially

abundant? 1: if in Healthy; -1: if in T2D; 0: not differentially abundant) (# of microbial species in the group) (species ID of the 1st microbial group member) (species ID of the 2nd microbial group member) …., (species ID of the last microbial group member)

Example:

1	0	5	1001	1005	1009	1012	1035
2	-1	4	1011	1034	1058	1063	

Note that group IDs should be distinguishable from species IDs.

3. List of *small-molecule metabolites*

File name: **small_molecule_info.txt**

Format: First row shows the total number of small-molecule metabolites shown in this file. From the second row and onward, each row shows the ID of each metabolite.

4. List of *macromolecules*

File name: macromolecule_info.txt

Format: First row shows the total number of macromolecules shown in this file. From the second row and onward, each row shows the ID of each macromolecule.

5. Information on the *microbial metabolic transport network (NJS16)*

File name: network_info.txt

Format: First row shows the total number of links in NJS16, along with some additionally generated links, i.e. link type 7 and 8 (below), derived from the contents of NJS16. From the second row and onward, each row corresponds to each link. Each link is in the format of (tab-delimited):

(metabolite ID) (species ID or metabolite ID) (link type)

The second column is a metabolite ID only if the corresponding link type is a 7. Link types are defined accordingly:

2: The metabolite is directly consumed (imported) by the microbe

3: The metabolite is directly produced (exported) by the microbe

5: The metabolite is both directly consumed (imported) and produced (exported) by the microbe

6: The macromolecule is subject to extracellular degradation by the microbe

7: The metabolite in the second column is derived from the extracellular degradation of the macromolecule in the first column (follows Supplementary Data 2g).

8: The metabolite is indirectly produced (exported) by the microbe via extracellular macromolecule degradation. In other words, this link consists of a microbe and a degradation product of the macromolecule. These links are provided simply for reference, and having these links as part of the

input file is not necessary as they are generated internally while running our program.

Algorithm for identifying metabolic influences between all ordered pairs of microbial entities

W_{ij} is calculated according to this formula:

$$W_{ij} \approx \sum_{k} [E_{ki} \cdot (\alpha \; or \; \beta) \; - F_{ki}]$$

Here, $E_{ki} \cdot (\alpha \; or \; \beta)$ and F_{ki} are defined as follows:

$$E_{ki} \cdot (\alpha \; or \; \beta) = \begin{cases} \frac{n_i}{\sum_x n_x} \alpha \; if \; N = 0 \\ \frac{n}{N} \beta \; if \; N > 0 \; and \; n_i \geq \theta_1 \\ \frac{\beta}{N} \sum_{PP_k^m} \frac{n_i}{\sum_{z \in P_k^m} n_z} \; if \; N > 0 \; and \; n_i < \theta_1 \end{cases}$$

$$F_{ki} = \frac{n_i}{\sum_y n_y}$$

For notations, please refer to Supplementary Information Sections 2.1 and 2.3.

We use the following three steps to obtain the W-matrix (explained in detail below):

1. Calculate E_{ki} and F_{ki} for each pair of small-molecule metabolite *k* and microbe *i*.
2. Calculate $\sum_k E_{ki}$ and $\sum_k F_{ki}$ for each pair of microbial entities *i* and *j*.
3. Calculate W_{ij}.

Step 1

In this step, we obtain three values for each metabolite *k* and microbe *i*: $\mathrm{B_k}$ (1 if *k* is derived from a macromolecule; 0 if otherwise), E_{ki} (the producer term of metabolite *k* and microbe *i*), and F_{ki} (the consumer term of metabolite *k* and microbe *i*).

For each metabolite *k*:

P_k: The set of microbes that directly export *k*;

P_k^m: The set of microbes that indirectly export *k* by degrading macromolecule *m*;

C_k: The set of microbes that directly import *k*;

N: # of macromolecules (*m*'s) satisfying $\sum_{z \in P_k^m} n_z \geq \theta_1$;

#Calculate consumer term

Initialize $F_{ki} = 0$ for every microbe *i*

$TotalConsPop = \sum_{y \in C_k} n_y$

For each consumer *i* of *k*:

If $TotalConsPop < \theta_1$, $F_{ki} = 0$ for all microbe *i*;

Else, $F_{ki} = n_i / TotalConsPop$ for each microbe *i*;

#Calculate producer term

Initialize $E_{ki} = 0$ for every microbe *i*

$TotalProdPop = \sum_{x \in P_k} n_x$;

For each macromolecule *m*:

$TotalPopMacro_m$;

If $N = 0$:

$B_k = 0$;

For each direct exporter *i* of *k*:

If $TotalProdPop < \theta_1$, $E_{ki} = 0$ for all microbe *i*;

Else, $E_{ki} = n_i / TotalProdPop$ for each microbe *i*

Else:

$B_k = 1$;

For each microbe *i* that indirectly exports *k*:

H_i: set of m's such that $\sum_{z \in P_k^m} n_z \geq \theta_1$ and *i* can degrade *m*;

n: # of elements in H_i;

If $n_i \geq \theta_1$:

$E_{ki} = \frac{n}{N}$;

Else:

$E_{ki} = \frac{1}{N} \sum_{m \in H_i} \frac{n_i}{TotalPopMacro_m}$ for *m*;

The result of **Step 1** is saved as "met_microbe_coupling.txt" in the "Output_files" directory.

Step 2

In this step, we calculate terms in W_{ij} that are independent of alpha and beta. Precisely, for two microbes i and j, $W_{ij} = A_{ij}\alpha + B_{ij}\beta + C_{ij}$. We will obtain A_{ij}, B_{ij}, and C_{ij} for any two microbes i and j and also for any microbe-group pair and any group-group pair.

We start with microbe-microbe pairs. For each microbe pair (*i, j*):

$A_{ij} = \sum_k E_{kj}$ for k such that i produces k and $B_k = 0$;

$B_{ij} = \sum_k E_{kj}$ for k such that i produces k and $B_k = 1$;

$C_{ij} = -\sum_k F_{kj}$ for k such that i directly consumes k;

For each ordered pair of (group *G*, microbe *j*):

$A_{Gj} = \sum_l A_{lj}$ for l in G;

$B_{Gj} = \sum_l B_{lj}$ for l in G;

$C_{Gj} = \sum_l C_{lj}$ for l in G;

For each ordered pair of (microbe *i*, group *G*):

$A_{iG} = \sum_l \frac{n_l}{n_G} A_{il}$ for l in G;

$B_{iG} = \sum_l \frac{n_l}{n_G} B_{il}$ for l in G;

$C_{iG} = \sum_l \frac{n_l}{n_G} C_{il}$ for l in G;

Here, n_G is the sum of the abundances of all microbial species in group *G*. If $n_G = 0$, then $\frac{n_l}{n_G}$ is replaced with 1/(# of species in *G*) for the 3 equations above.

For each ordered pair of (group *G*, group *Γ*):

$A_{G\Gamma} = \sum_{l,q} \frac{n_q}{n_{\Gamma-G}} A_{lq}$ for l in G and q in Γ-G (set difference of Γ and G);

$B_{G\Gamma} = \sum_{l,q} \frac{n_q}{n_{\Gamma-G}} B_{lq}$ for l in G and q in Γ-G (set difference of Γ and G);

$C_{G\Gamma} = \sum_{l,q} \frac{n_q}{n_{\Gamma-G}} C_{lq}$ for l in G and q in Γ-G (set difference of Γ and G);

If $n_{\Gamma-G} = 0$, then $\frac{n_q}{n_{\Gamma-G}}$ is replaced with 1/(# of species in Γ-G) for the 3 equations above.

The result of **Step 2** is saved as “node_node_ABC.txt” in the “Output_files” directory.

Step 3

In this step, we finally calculate the W-matrix with alpha = 1.0 and beta=1.0 (these values for alpha and beta were used in our analysis). We use the formula $W_{ij} = A_{ij}\alpha + B_{ij}\beta + C_{ij}$ for every pair of microbial entities.

The result of this step is saved as “Wmatrix_a1.0_b1.0_thre1e-4.txt” in the “Output_files” directory.

This marks the end of our algorithm for our metabolic influence computation.
